# Neuroblastic tumors and neurofibromatosis type 1: A retrospective multicenter study in Italy and systematic review of the literature

**DOI:** 10.3389/fped.2022.950911

**Published:** 2022-11-04

**Authors:** Federica Puglisi, Rachele Soma, Marta Podda, Simona Vetrella, Marco Rabusin, Serena Tropia, Mariaclaudia Meli, Giovanna Russo, Stefania Sorrentino, Giovanni Erminio, Alfredo Pulvirenti, Martino Ruggieri, Andrea Di Cataldo

**Affiliations:** ^1^ Unit of Neonatology and Neonatal Intensive Care Unit, AOU “Policlinico”, PO “San Marco”, University of Catania, Catania, Italy; ^2^ Unit of Pediatric Onco-Haematology, Department of Clinical and Experimental Medicine, Section of Pediatrics and Child Neuropsychiatry, University of Catania, Catania, Italy; ^3^ Pediatric Oncology Unit, Fondazione IRCCS Istituto Nazionale Tumori, Milan, Italy; ^4^ Pediatric Oncology Unit, Santobono-Pausilipon Hospitals, Naples, Italy; ^5^ Institute for Maternal & Child Health (I.R.C.C.S) Burlo Garofolo, Trieste, Italy; ^6^ Pediatric Hematology and Oncology Unit, ARNAS “Civico, Di Cristina and Benfratelli” Hospitals, Palermo, Italy; ^7^ Oncology Unit, IRCCS Istituto Giannina Gaslini, Genova, Italy; ^8^ Epidemiology Scientific Directorate, IRCCS Istituto Giannina Gaslini, Genova, Italy; ^9^ Bioinformatics Unit, Department of Clinical and Experimental Medicine, University of Catania, Catania, Italy; ^10^ Unit of Rare Diseases of the Nervous System in Childhood, Department of Clinical and Experimental Medicine, Section of Pediatrics and Child Neuropsychiatry, University of Catania, Catania, Italy

**Keywords:** neurofibromatosis type 1, NF1, neuroblastoma, child, cancer

## Abstract

**Background:**

Neuroblastic tumors (NBTs) are the most common extra-cranial solid tumors of childhood. Neurofibromatosis type 1 (NF1) is the most common neurocutaneous disorder with a predisposition to tumors. The co-occurrence of NBTs in the setting of NF1 has been occasionally reported, suggesting a non-casual association and likely configuring a spectrum of neural crest–derived disorders.

**Aim of the study:**

To explore the occurrence of NBTs within NF1 and to report on its natural history, therapeutic strategies, and outcomes in an Italian cohort of children with NF1 and in the literature.

**Subjects and Methods:**

Study (a): a retrospective analysis of questionnaire-based data [years 1979–2017] derived from the databases of the Italian Registry for Neuroblastoma (RINB) of the Italian Society of Pediatric Onco-Haematology (AIEOP); and Study (b): a systematic review search on NF1/NB co-occurrence.

**Results:**

Study (a) identified eight children with NBTs, 0.2% of patients registered in the RINB, fulfilling the diagnostic criteria for NF1. The primary site of NBTs was abdominal in six patients. The NBTs were neuroblastoma (NB) in five patients, ganglioneuroblastoma (GNB) in one, patient, and ganglioneuroma (GN) in two. Metastatic diffusion occurred in three out of eight children. *MYCN* gene testing, performed in the tumors of five patients, resulted not-amplified. The major features of NF1 included the following: NF1 family history in four patients, café-au-lait spots in all, freckling in six, Lisch nodules in three, and neurofibromas in three. With regard to the outcome, four children survived three of these for the progression of NB and one for a second tumor. Study (b) identified 12 patients with NF1/NB from the years 1966–2017, and the median age at diagnosis was 27 months (range = 0–168 months). The primary site of NB was thoracic. The prevalent histotype was NB in nine patients, GNB in two, and GN in one. Eight/nine NBs were metastatic. The *MYCN* gene was amplified in the only studied case. The NF1 features included NF1 family history in seven patients; the major NF1 features were café-au-lait spots in nine patients, freckling in one, Lisch nodules in none, and neurofibromas in six. The outcome was good for only two children, while eight children died of neuroblastoma, at a median age of 49.5 months (range = 2.4–174 months), with a median survival time of 21.75 months after diagnosis.

**Conclusions:**

To our knowledge, this represents the first systematic study on the occurrence of NBTs in NF1. This confirms that NBs are rare *per se* in the setting of NF1 (0.2% of all NBs) and even if compared to the overall frequency of malignancies in NF1 (i.e., 14.7%). The male:female ratio in study (a) (0.6) was different from what was recorded in study (b) (1.5) and in line with the overall increased frequency of malignancies in females with NF1. The median ages at diagnosis of NB in either study (a) or (b) were concordant with what occurred in the NB population. In study (a) versus study (b), the frequency of metastatic diffusion was lower, likely indicating less awareness on work-ups for malignancies in old NF1 series in the literature. The outcome was much better in study (a) than in study (b), indicating that multidisciplinary treatment for NB is highly recommended.

## Introduction

Neuroblastic tumors (NBTs) are very rare tumors, characterized by various clinical presentations and diverse prognosis of its subsets. While in some patients the tumor is successfully treated with surgery alone, or may regress spontaneously, the chances of cure in children older than 1 year with metastatic disease remain poor. Although the probability of survival for children with NBTs has improved over time, even the best published results do not parallel those obtained for most other childhood malignancies (1). A few studies have documented this finding by reporting a large series of patients diagnosed over a long period (2–6).

Most NBTs are sporadic and not correlated with any specific constitutional germline chromosomal abnormality, inherited predisposition, or associated congenital anomalies. Nevertheless, there are some exceptions. A higher incidence of NBTs has been suggested in girls with Turner syndrome (7). Patients with Kabuki syndrome and NBTs have been reported (8, 9). Hirschsprung’s disease, congenital central hypoventilation (Ondine's curse), and neurofibromatosis type 1 (NF1) have all been described in association with NBTs, suggesting the existence of a global disorder of neural crest–derived cells (i.e., neurocristopathy) (10–13).

NF1 is the most common form of neurofibromatosis and one of the most common autosomal dominant disorders in humans, with an incidence of 1 in 2,600–3,000 individuals (14, 15). Approximately one half of the cases are familial (inherited). The remainder are the result of *de novo* (sporadic) mutations (16). These mutations occur primarily in paternally derived chromosomes, and the likelihood of *de novo* NF1 increases with advanced paternal age (17). The incidence of segmental NF1 is estimated at 1 in 36,000–40,000 (18).

Patients with NF1 are predisposed to both benign and malignant tumors of neurogenic and non-neurogenic origin. Most studies that have addressed the risk of malignancy and early death have shown an approximately 8- to 15-year decrease in life expectancy in patients with NF1, mainly as a result of malignancy (19, 20). Mortality in NF1 has previously been studied in cohorts from France, Wales, United States, and Denmark (21–23). These studies have shown excess mortality rates of NF1 patients compared with those of the general population and a high proportion of deaths caused by malignancies.

Multiple studies have shown a substantial risk of nervous system malignancy, with an indisputable excess risk of gliomas and malignant peripheral nerve sheath tumor (MPNST) (24), both of which result in an excess risk of mortality (25). The onset of NBTs in patients with NF1 has also been described, but the actual incidence of this association is not known (26).

The purpose of this study is to analyze the clinical–epidemiological characteristics of this rare clinical association, as well as the therapeutic approach and prognosis, in order to allow a descriptive analysis of the development of clinical and research experience.

## Materials and methods

### Setting and sample

All patients with a diagnosis of a NBT and NF1 were included in this analysis. The cases were identified by searching the databases of the Italian clinical units of pediatric onco-hematology and the Italian Neuroblastoma Registry (RINB) database (from 1979 onward). The RINB was activated in 1979 and includes all subjects with any peripheral NBT [i.e., neuroblastoma (NB), ganglioneuroblastoma (GNB), and the benign ganglioneuroma (GN)], diagnosed at the institutions included in the Italian Neuroblastoma Group (ING) of the Italian Association of Pediatric Hematology and Oncology (AIEOP) (27). More than the expected number of NBT patients from Italy have been recruited through this network (28).

The data on the included patients were collected retrospectively: patients diagnosed from the years 1979 to May 2017 were considered. The patients were treated according to the AIEOP protocol till the 1990s and European SIOPEN protocols thereafter. Informed consent to the treatment and to data collection and analysis was obtained from all patients according to institutional guidelines at the time of enrolling patients in the protocols.

A standardized questionnaire was sent to all the 54 hematology-oncology units belonging to the AIEOP with the aim of collecting information about the two conditions. The survey was designed to assess data regarding epidemiological aspects, clinical features, and genotype/phenotype correlation and the management and course of the patients individuated. The questionnaire included two parts, one relating to a description of the NBT and the other relating to NF1, as given in Tables 1, 2. Tumor diagnosis, disease extension, and response to treatment were defined according to the International Neuroblastoma Staging System (INSS) and the International Neuroblastoma Risk Group (INRG) staging system as described previously (29, 30). The long-term sequelae were ascertained by contacting the clinical investigators at the various centers; no additional investigations were conducted on possible late effects for the purposes of this study.

**Table 1:** Characteristics of the tumor in eight patients.

Patient No.	1	2	3	4	5	6	7	8
Clinical presentation								
Sex	Male	Female	Female	Male	Female	Female	Female	Male
Age at diagnosis (months)	30	68	92	57	23	24	120	24
Symptoms at diagnosis	No	Dysuria and vulva edema	No	Abdominal pain	No	Abdominal distension	Rapidly growing neurofibroma	No
Primitive tumor site	Abdomen (adrenal gland)	Pelvis	Abdomen (adrenal gland)	Abdomen (adrenal gland)	Pelvis	Abdomen (ganglia)	Abdomen (ganglia)	Abdomen (adrenal gland)
Histology	NB (NOS)	GN	NB (NOS)	NB (NOS)	GN	NB (NOS)	GNB nodular	NB (NOS)
Diagnosis procedure	Biopsy	Biopsy	Biopsy	Bone marrow aspirate	Biopsy	Biopsy	Biopsy	Biopsy
Dumbbell tumor	No	No	No	No	Unknown	No	No	No
Staging								
INSS	2A	NA	4	4	NA	4	3	1
INRG	L1	NA	M	M	NA	M	L2	L1
Metastases	No	NA	distant lymph nodes, bone	bone marrow, bone, distant lymph nodes, and orbit	NA	bone marrow	No	No
Biochemical data								
Serum LDH	Normal	Normal	Elevated	Elevated	Normal	Elevated	Normal	Elevated
Urine VMA	Unknown	Normal	Elevated	Unknown	Unknown	Elevated	Normal	Unknown
Serum Ferritin	Unknown	Normal	Unknown	Unknown	Unknown	Normal	Unknown	Unknown
Molecular data at onset								
*MYCN* status	Normal	Not Done	Normal	Not Done	Not Done	Normal	Normal	Normal

GN, ganglioneuroma; GNB, ganglioneuroblastoma; INRG, International Neuroblastoma Risk Group Staging System; INSS, International Neuroblastoma Staging System; LDH, lactate dehydrogenase; NA, not applicable; NB, neuroblastoma; NOS, not otherwise specified; VMA, vanillylmandelic acid.

**Table 2:** Characteristics of NF1 in eight patients.

Patient	1	2	3	4	5	6	7	8
Sex	Male	Female	Female	Male	Female	Female	Female	Male
Age at diagnosis (months)	15	30	84	8	12	24	120	8
Pregnancy	Unknown	Unknown	Normal	Normal	Unknown	Normal	Normal	Macrosomia
* *NF1 family history	Unknown	Mother	No	No	Mother Brother	Father	No	Father
Perinatal period	Phototherapy for jaundice	Unknown	Normal	Normal	Patent foramen ovale	Acute pyelonephritis	Normal	Normal
*NF1 features*								
*Major features*								
Café-au-lait spots								
Trunk	Yes	Yes	Yes	Yes	Yes	Yes	Yes	Yes
Arms	Yes	Yes	Yes	Yes	Yes	Yes	Yes	Yes
Legs	Yes	Yes	Yes	Yes	Yes	Yes	Yes	Yes
Freckling								
Axillae	Yes	Yes	Yes	Unknown	Yes	Yes	Yes	No
Groin	Yes	No	Yes	Unknown	Yes	Yes	Yes	No
Neck	No	No	Yes	Unknown	Yes	Yes	No	No
Perioral	No	No	No	Unknown	No	No	No	No
Lisch nodules	Bilateral	Unknown	Bilateral	Unknown	No	No	No	Monolateral
Neurofibromas								
Trunk	No	No	No	No	Yes	No	Yes	No
Arms	No	No	No	No	Yes	No	Yes	No
Legs	Yes	No	No	No	Yes	No	Yes	No
*Minor features*								
Short stature	No	Yes	Yes	Unknown	No	No	No	No
Head circumference	Macrocephaly	Normal	Macrocephaly	Macrocephaly	Unknown	Macrocephaly	Normal	Macrocephaly
Dysmorphic features	No	No	Yes	Yes	Yes	No	Lumbar scoliosis	No
*Complications*	Unknown	No	Optic glioma	Unknown	Bilateral Hydronephrosis (with nephrostomies) Pelvic plexiform neurofibromas Clitoral hypertrophy	No	No	No
Cognitive profile	Normal	Normal	Normal	Cognitive deficit	Unknown	Unknown	Normal	Normal
Behavioral profile	Unknown	Normal	Normal	Psychomotor deficit	Unknown	Hyperactivity	Normal	Normal
Brain MRI	Hamartoma	Unknown	Optic glioma	Not done	Optic glioma UBOs	Not done	UBOs	Not done
Spinal MRI	Not done	Unknown	Normal	Not done	Neurofibromas with spinal cord invasion and compression, MPNST	Not done	Hydromyelia	Not done
NF1 gene analysis	Unknown	Not done	Not done	Unknown	Positive	Positive	Positive	Positive

MPNST, malignant peripheral nerve sheath tumor; MRI, magnetic resonance imaging; UBOs, unidentified bright objects.

Statistical analysis was conducted within the R statistical System. To identify possible correlations among the subgroup of patients, we performed principal component analysis using the variables characterizing both NF1 and NBTs.

## Results

### Demographic information

Overall, eight patients with NBTs and NF1 were registered, who included three males and five females, with a male:female ratio of 0.6. They represented 0.2% of all patients registered in the RINB (3,976 patients). The age at diagnosis of neuroblastoma was between 23 and 120 months, with an average of 54.7 months and a median age of 43.5 months. The age at diagnosis of NF1 was between 8 and 120 months, with an average of 37.6 and a median age of 19.5 months. The patients’ demographic data are given in Table 3.

**Table 3:** Demographic information of the study patients.

Characteristic	All patients (*n* = 8)
Sex, No.	
Male	3
Female	5
Age in months at diagnosis of NB	
Mean	54.7
Median	43.5
Range	23–120
Age in months at diagnosis of NF1 (evaluated only in six patients)	
Mean	37.6
Median	19.5
Range	8–120

### Clinical presentation

The abdomen was the primary site for six patients, the adrenal gland for four patients, paravertebral ganglia for two, and pelvis for two. Symptoms at diagnosis were present in half of the patients and depended on the primary site: one child had dysuria and vulva edema, two had abdominal pain, and only one patient had a symptom correlated with NF1, an increase in the size of a neurofibroma in the same site. No patient had a dumbbell tumor (Tables 1, 2).

The histology most represented was NB in five patients. One patient had a GNB nodular, and two had GN. Among these five patients with neuroblastoma, metastatic disease was present at onset in three patients; the other two cases with NB,were both stage L1 for the INRG and stage 2A and 1 for the INSS. The sites of metastasis were distant lymph nodes, bone, and bone marrow in two patients and orbit in one.

With regard to biologic studies at diagnosis, serum lactate dehydrogenase (LDH) was studied in all patients and was found to be above normal value in four patients; serum ferritin was normal in the two studied cases, while urinary vanillylmandelic acid (VMA) was above normal value in two of the four studied cases. The *MYCN* gene was not amplified in the five studied cases.

In six patients, NF1 was diagnosed before the diagnosis of NBTs, while in two patients, it was diagnosed at the same time. Half of the patients had a family history of NF1. The perinatal period was uneventful for four patients, one was treated for jaundice at birth, one was treated for urinary tract infection, and one had a patent foramen ovale at birth. At diagnosis, café-au-lait spots were found on all patients, distributed over the trunk, arms, and groin in all of them; freckling was recorded in seven patients; bilateral ocular Lisch nodules were detected in two patients and monolateral ocular Lish nodules in one. Three children developed neurofibromas. For what was regarded as minor features, macrocephaly was reported in five patients, short stature in two, and dysmorphic features in four. With regard to the cognitive and behavioral profile, one case of hyperactivity was reported and there was one case of cognitive and psychomotor deficit. On brain magnetic resonance imaging (MRI), optic glioma was detected in two patients and hamartoma in one; unidentified bright objects (UBOs) were observed in two. Furthermore, spinal MRI showed the presence of neurofibromas with a compression of the spinal cord in one patient and hydromyelia in another. Genetic testing was performed in four patients to confirm the diagnosis. Patient No. 5 was the one who presented the most significant number of complications related to NF1, with the presence of bilateral hydronephrosis (with nephrostomies), clitoral hypertrophy, pelvic plexiform neurofibromas, neurofibromas with invasion of the spinal canal, and a compression of the spinal cord and MPNST.

Because of the availability of only a few samples, we were not able to establish any statistically significant relationship among these patients.

### Treatment

Treatment and outcome are summarized in Table 4. As first-line treatment, surgery was performed on two patients, and it involved a complete resection. No postoperative complications were reported. Four patients received multidrug chemotherapy according to their time protocols. Only one patient received, after induction chemotherapy, a consolidation treatment with megatherapy and stem cell rescue, radiotherapy, immunotherapy, and cis-retinoic acid. Only one patient underwent 131I-metaiodobenzylguanidine (MIBG) therapy, given the intense capture of the documented mass with MIBG scintigraphy.

**Table 4:** Treatment details and outcome for eight patients.

Patient no	1	2	3	4	5	6	7	8
Treatment								
Surgery first line	Complete resection	No	No	No	No	No	No	Complete resection
Chemotherapy first line	No	No	Yes	Yes	No	Yes	Yes	No
Megatherapy with stem cell rescue	No	No	Yes	No	No	No	No	No
Radiotherapy first line	No	No	Yes	No	No	No	No	No
Therapeutic MIBG	No	No	No	No	Yes	No	No	No
Immunotherapy	No	No	Yes	No	No	No	No	No
Cis-retinoic acid	No	No	Yes	No	No	No	No	No
Response to treatment and outcome								
Response	CR	SD	DP	DP	DP	DP	CR	CR
Relapse/Disease Progression	No	No	DP After first-line therapy	DP During first-line therapy	DP after MIBG therapy	DP After first-line therapy	No	No
Rescue treatment	No	No	Chemotherapy	Chemotherapy	Chemotherapy	Chemotherapy	No	No
Outcome (Months after diagnosis)	Alive CR (44,5)	Alive SD (96)	Death from DP (19)	Death from DP (6)	Death caused by MPNST (unknown)	Death from DP (20)	Alive CR (7)	Alive CR (53)

CR, complete response; DP, disease progression; MIBG, 131I-metaiodobenzylguanidine; MPNST, malignant peripheral nerve sheath tumor; SD, stable disease.

### Outcome

Three patients reached the stage of complete remission (CR) after first-line treatment, two reached the surgery alone stage, and one the chemotherapy stage, and these patients are in CR at 44.5, 53, and 7 months, respectively, after diagnosis; only one patient was observed and is in a condition of stable disease (SD) 8 years after diagnosis. Disease progressed in four patients after first-line therapy in two, during first-line therapy in one, and after MIBG therapy in one. All of them received rescue chemotherapy, but they all died, three of NB at 6, 19, and 20 months from diagnosis and one from a second tumor (mediastinal MPNST), but their time of death was not reported.

## Literature review and discussion

**Figure 1:**
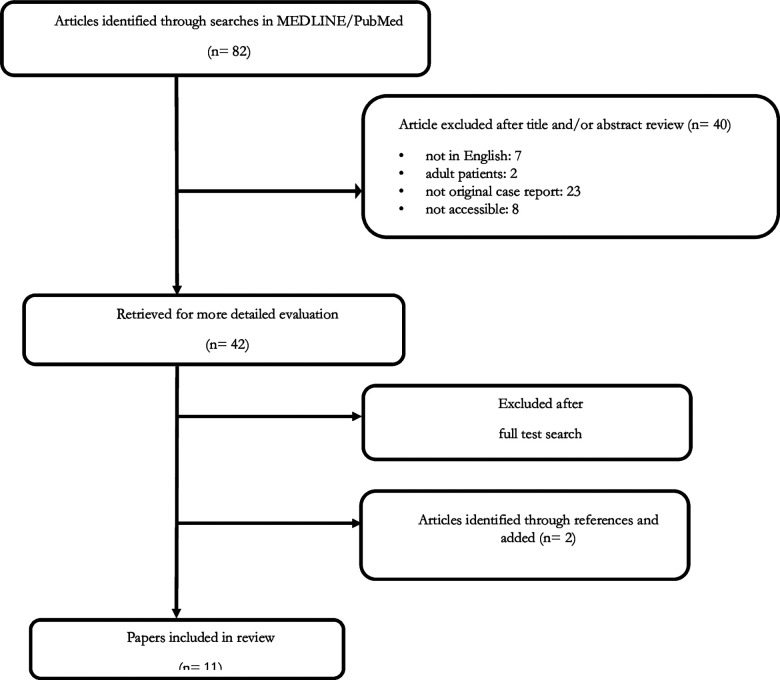
Selection process for papers included in the review.

A literature study was performed using MEDLINE/PubMed up to May 2017. The following MeSH headings and text words were used: “neuroblastoma AND neurofibromatosis type 1” and “neuroblastoma AND von Recklinghausen disease”. We did not impose any language restrictions. Two additional articles from the reference lists were obtained. Finally, all papers identified in the literature scan were examined and relevant papers were reviewed and summarized for inclusion in this report (31–38) (Figure 1).

Overall, 12 cases of patients with NBTs and NF1 were reported between 1966 and 2013: 7 males and 3 females (the sex of the other two patients was not available). The age at diagnosis of NBTs was between 0 and 168 months, with an average of 39 months and a median age of 27 months. The age at diagnosis of NF1 was specified only in five patients, while in three patients, it was not reported, and in four patients, NF1 was diagnosed at the same time of NB diagnosis. The patients’ demographic data are presented in Table 5.

**Table 5:** Demographic information of the case reports.

Characteristic	Reported only on 10 patients
Sex, No. Male	7
Female	3
Not available	2
Age in months at diagnosis of NBT	
Mean	39.5
Median	27
Range	0–168
Age in months at diagnosis of NF1 (evaluated on nine patients)	
Mean	23.1
Median	14
Range	2–72
Age at death	
Mean	76.3
Median	49.5
Range	2.4–174
Survival in months after diagnosis	
Mean	38.8
Median	21.75
Range	0–120

NBT, neuroblastic tumor.

A detailed description of the case reports is given in Tables 6A,B. The primary site of NBTs was known in 11 out of 12 patients. It was in the mediastinum in five patients, in the abdomen in four patients, and in the neck in one, and in one patient, there were two masses, both in the thorax and in the abdomen. Diagnostic symptoms were present in all patients and correlated with the primary site. Dumbbell tumor was found in two patients, one with NB and one with GNB.

**Table 6A:** Description of the cases reports analyzed by the literature (part 1).

Patient No.	1	2	3	4	5	6
Author, tear	Cheuk, 2013	Duhem-Tonnelle, 2006	Origone, 2003	Martinsson, 1997	Hayflick, 1990	Qualman, 1986
Year	2013	2006	2003	1997	1990	1986
Sex	Male	Male	Male	Unknown	Male	Female
Age at diagnosis of NBT (months)	19	22	36	72	9	2 (autopsy)
Age at diagnosis of NF1 (months)	unknown	unknown	36	72	9	2 (autopsy)
NF1 family history	unknown	Positive (not specified)	mother and brother	Father	father	No
NF1 features described	unknown	CAL	CAL	CAL	CAL, NF	CAL, NF
*NF1* gene analysis	Unknown	Unknown	Positive	Positive	Unknown	Unknown
Symptoms at diagnosis	Supraclavicular lymphadenopathy hepatomegaly	Progressive tetraparesis associated with cervical pain	Paleness, periorbital ecchymosis, and a large abdominal mass	Unknown	Right arm and bilateral lower-extremity weakness edema of the right arm	Apnea, cyanosis
Associated clinical conditions	Unknown	Unknown	tibial pseudoarthrosis	Unknown	short stature, retarded psychomotor development	Schwannoma EEG abnormalities Macroglossia Laryngomalacia Meckel's diverticulum esophageal gastric heterotopia
Primitive tumor site	unknown	Neck	Abdomen (ganglia)	Thorax	Thorax	Abdomen (bilateral adrenal gland)
Histology	NB	GNB	NB	GNB	NB	NB
Diagnosis procedure	Unknown	partial resection with laminotomy	Bone marrow aspiration and MIBG	complete resection	Biopsy	Autopsy
Dumbbell tumor	Unknown	Yes	No	No	Yes	No
INSS	4	Unknown	4	2A	4S	unknown
Metastases	Yes (site no specified)	No	Liver Bone marrow	No at diagnosis Bone and bone marrow at relapse	Lymph nodes Lungs Neck	No
Serum LDH	Unknown	Unknown	elevated	Unknown	Unknown	Unknown
Urine VMA	Unknown	Unknown	elevated	Normal (elevated at relapse)	elevated	Unknown
*MYCN* status	Unknown	Unknown	Amplified	Unknown	Unknown	Unknown
Surgery first line	Yes	Yes	No	Yes	Yes	No
Chemotherapy first line	Yes	No	Yes	No	Yes	No
Radiotherapy first line	Yes	No	No	No	Unknown	No
MIBG	Unknown	Unknown	Unknown	Unknown	Unknown	No
Response	CR	PR	CR	CR	NR	DP
Relapse/disease progression	Relapsed at 15 months	DP 2 years after diagnosis	Relapsed at 13 months after CR	Relapsed at 2,5 years after CR	Unknown	None
Rescue treatment	Unknown	Surgery	Unknown	S, CT, RT, MIBG, and retinoic acid	Unknown	None
Outcome	Died at 39 months for DP (20 months after diagnosis)	Died at 108 months for respiratory failure (84 months after diagnosis)	Died at 60 months (26 months after diagnosis)	Died at 168 months (96 months after diagnosis)	Unknown	Died at 2 months for disease (aspiration pneumonia and partial airway obstruction)

CAL, cafè-au-lait spot; CR, complete response; CT, chemotherapy; DP, disease progression.; GNB, ganglioneuroblastoma; INSS, International Neuroblastoma Staging System; LDH, lactate dehydrogenase; MIBG, 131I-metaiodobenzylguanidine; NB, neuroblastoma; NBT, neuroblastic tumor; NF neurofibroma; NR not reported; PR, partial response; RT, radiotherapy; S, surgery; VMA, vanillylmandelic acid.

**Table 6B:** Description of the cases reports analyzed by the literature (part 2).

Patient No.	7	8	9	10	11	12
Author, year	Qualman, 1986	Kushner, 1985	Nakagawara, 1985	Weiner, 1982	Witzleben, 1974	Knudson, 1966
Year	1986	1985	1985	1982	1974	1966
Sex	Male	Unknown	Female	Male	Male	Female
Age at diagnosis of NBT (months)	6	54	168	54	32	0
Age at diagnosis of NF1 (months)	3	24	14	NR	12	36
NF1 family history	No	Mother	Brother	Unknown	mother, father	No
NF1 features described	NF	CAL, freckling	CAL, NF	Unknown	CAL, NF	CAL, NF
NF1 gene analysis	Unknown	Unknown	Unknown	Unknown	Unknown	Unknown
Symptoms at diagnosis	Abdominal masses and nasopharyngeal tumor	Fever Leg pains	Fever, abdominal mass, weight loss, ptosis	Fatigue, epistaxis, leg pains, hepatosplenomegaly, lymphadenopathy	Lethargy, irritability, somnolence, cachectic, abdominal mass	Persistent vomiting
Associated clinical conditions	Schwannoma NF	Unknown	Malignant pheochromocytoma	LLA	Short stature, Optic glioma	Clitoris hypertrophy, Horner's syndrome
Primitive tumor site	Thorax, abdomen	Abdomen (adrenal gland)	Abdomen (adrenal gland)	Thorax	Thorax	Thorax
Histology	NB	NB	NB	GN	NB	NB and GN
Diagnosis procedure	Biopsy	unknown	Biopsy (liver metastasis)	Resection	Resection	Biopsy (liver metastasis)
Dumbbell tumor	Unknown	Unknown	Unknown	No	No	unknown
INSS	4	4	4	NA	4	4s
Metastases	Lungs, prostate	Bone, bone marrow	Liver, bone, lungs, lymph nodes, bone marrow	Unknown	Bone marrow, liver, lungs, lymph nodes, bone	Liver
Serum LDH	Unknown	Unknown	Elevated	Unknown	Unknown	Unknown
Urine VMA	Normal	Unknown	Elevated	Normal	Unknown	Unknown
MYCN status	Unknown	Unknown	Unknown	Unknown	Unknown	Unknown
Surgery first line	Yes	Unknown	Yes	Yes	Yes	Yes
Chemotherapy first line	Yes	Unknown	Yes	No	Yes	Yes
Radiotherapy first line	Yes	Unknown	Unknown	Unknown	Yes	Yes
MIBG	Unknown	Unknown	Unknown	Unknown	Unknown	Unknown
Cis-retinoic acid	Unknown	Unknown	Unknown	Unknown	Unknown	Unknown
Response	DP	NR	DP	CR	DP	PR
Relapse/disease progression	Unknown	Unknown	Unknown	Unknown	Unknown	No
Rescue treatment	Unknown	Unknown	Unknown	Unknown	unknown	No
Outcome	Died at 26 months (23,5 months after diagnosis) for progressive airways obstruction and portal hypertension	unknown	Died at 174 months (6 months after the diagnosis)	Alive at 12 months after diagnosis	Died at 33 months (1 month after the diagnosis)	Alive in CR at 120 months from diagnosis

CAL, cafè-au-lait spot; CR, complete response; DP, disease progression.; GN, ganglioneuroma; INSS, International Neuroblastoma Staging System; LDH, lactate dehydrogenase; MIBG, 131I-metaiodobenzylguanidine; NA, not applicable; NB, neuroblastoma; NF neurofibroma; PR, partial response; VMA, vanillylmandelic acid.

The histotype was NB in nine patients, GNB in two, and GN in one. One diagnosis of NB (patient 12) was done on liver biopsy, while the primary thoracic tumor, whose histology was analyzed some months later, was a ganglioneuroma, probably due to the maturation of a NB. Among these nine patients with NB, eight were metastatic. In one patient with GNB, metastasis appeared at relapse in the bone and bone marrow.

The results of the biologic studies were reported only for six patients. Above normal values were recorded in two patients for LDH and in three for VMA. An *MYCN* gene study was performed in only one patient and it was amplified.

Seven patients had an NF1 family history. At diagnosis, cafe-au-lait spots were present on nine patients, and freckling was recorded in one. Lisch nodules were not detected in any patient. Six patients developed neurofibromas. With regard to the associated clinical conditions, short stature was reported in two patients, and tibial pseudarthrosis and clitoris hypertrophy, respectively, in one. One patient presented with macroglossia, laryngomalacia, Meckel's diverticulum, and esophageal gastric heterotopia. Two cases of schwannoma and one case of optic glioma were registered. Macrocephaly was not described. With regard to the cognitive and behavioral profile, only one case of retarded psychomotor development was reported. Genetic testing was performed in two patients.

A diagnostic biopsy was performed on four patients, two for primary and two for liver metastases, while a major resection was obtained in four patients, in one, together with laminotomy; in one patient, diagnosis was made by using a bone marrow aspirate and MIBG scintigraphy and in one patient after autopsy.

Among 11 patients whose treatment was reported, three received only chemotherapy during the first-line treatment and four both chemotherapy and radiotherapy. For patients with GN and GNB, only surgery was the first-line treatment. Patient No. 4 received chemotherapy, radiotherapy, retinoic acid, and therapeutic MIBG at relapse. Patient No. 1 was the only one who received, in addition to chemotherapy during the first-line treatment, megatherapy with stem cell rescue.

The outcome was not reported for two patients. Four patients survived after first-line therapy, in CR, but only one maintained CR and was described as “alive” 12 months after diagnosis, while the other three eventually relapsed and died of the disease. Two patients obtained a PR, but only one is now alive in CR, while the other relapsed and died of NB. The remaining four patients showed rapid progress but eventually died of NB. The median age at death was 49.5 months (range, 2–174 months), with a median survival of 21.75 months after diagnosis.

To the best of our knowledge, this is the first study on the spectrum of NBT in Italian patients with NF1, which is one of the cancer-predisposing genetic disorders. To provide additional information, we reviewed the clinical and survival data of children with NBT and NF1 enrolled in the RINB over a 38-year period.

The frequencies of benign and malignant tumor development are increased in children with NF1 compared with the healthy population. In a recent study, it was found that the incidence of malignancy in children with NF1 younger than 16 years was 14.7% (39). Our report confirms that NBTs are very rare in patients with NF1 (only 0.2% of all patients registered in the RINB). In most NF1-related malignancies, including astrocytomas, MPNSTs, and neuroblastomas, a biallelic mutation of the *NF1* gene function is found in the affected cells (40–43) demonstrated homozygous inactivation of the *NF1* gene found in NB cells of a patient affected by familial NF1 and stage 4 NB. It is known that patients with stage 4 NB display an aggressive behavior, which is possibly connected with genetic abnormalities like *MYCN* gene amplification, chromosome 1p36 deletion, and chromosome 17q gain (44). Furthermore, an analysis of the *NF1* gene in NB cells revealed a somatic paternal allelic deletion encompassing the introns 26 and 27b and a maternal-derived constitutional T → C transition in the donor splice site of intron 14. Martinsson et al. (43) described a homozygous deletion of the *NF1* gene in a patient affected by NB. This patient showed a large biallelic deletion of *NF1* in the tumor cells that also revealed chromosome 1p36 deletion but not *MYCN* amplification (43).

Although this particular patient had a prognostically favorable localized disease (stage 2), his tumor underwent progression, leading (like in our own case) to his death. This suggests that the mutation of the *NF1* gene may be associated with a more aggressive tumor behavior. Hence, homozygous inactivation of the *NF1* gene could result in a partial or total abrogation of neurofibromin activity, which would lead to increased intracellular RAS signaling; this could lead to abnormal cell proliferation. A maternal or paternal germline mutation associated with a paternal or maternal somatic deletion found in NB cells indicate that an inactivation of the *NF1* gene in tumor cells occurred according to the “two-hit” model. Moreover, this mutation could be also inherited in a *de novo* way. The two-hit hypothesis has also been suggested by Martinsson et al. to explain the homozygous inactivation of the *NF1* gene. A somatic inactivation of the still functioning *NF1* allele is believed to be required for tumor formation. This “second hit” creates a reduced function of neurofibromin in the affected cells, diminishing its normal functions, including those of controlling cell growth and proliferation. With this role of the *NF1* gene as a tumor suppressor, it is not surprising that somatic mutations of the *NF1* gene are also commonly found in different non-NF1-associated tumors. Both patients died of tumor progression, even though one had favorable clinical characteristics, suggesting that the biallelic inactivation of *NF1* might induce a more aggressive growth behavior of the disease.

Both in our study and in our review, the media and median age at diagnosis of neuroblastoma are similar, which is in agreement with the data that 95% of all neuroblastomas occur in children under 5 years of age. However, there is a difference in terms of the male-to-female ratio. In the review study, there is a prevalence of males, which is in concordance with the majority of reviewed studies in which neuroblastoma is found to be slightly more common among boys than among girls. However, the cases analyzed in the Italian study show a slightly greater frequency in females. This is in agreement with some studies that reported that females with NF1 had a higher risk of malignancy than males (26), but we did not replicate this observation, which may be attributed to our relatively small sample size limiting the statistical power to detect a difference.

Neuroblastomas have a very broad spectrum of clinical behavior, which can include spontaneous regression, maturation to a benign GN, or aggressive disease, with metastatic dissemination leading to death. This pattern of malignancies was similar to what we found in our NF1 patients and included a variety of different histotypes with a general prevalence of neuroblastoma on ganglioneuroblastoma and ganglioneuroma. Diagnostic dilemmas may arise in patients with NF1 and malignancy because neurofibroma and neuroblastoma share a common origin in the neural crest. Both show rapid growth and extensive invasion. Diagnosis is further complicated by the potential for different histologic components existing within one tumor mass. The biopsy of a portion of a heterogeneous tumor may not be representative of the spectrum of pathology.

**Figure 2:**
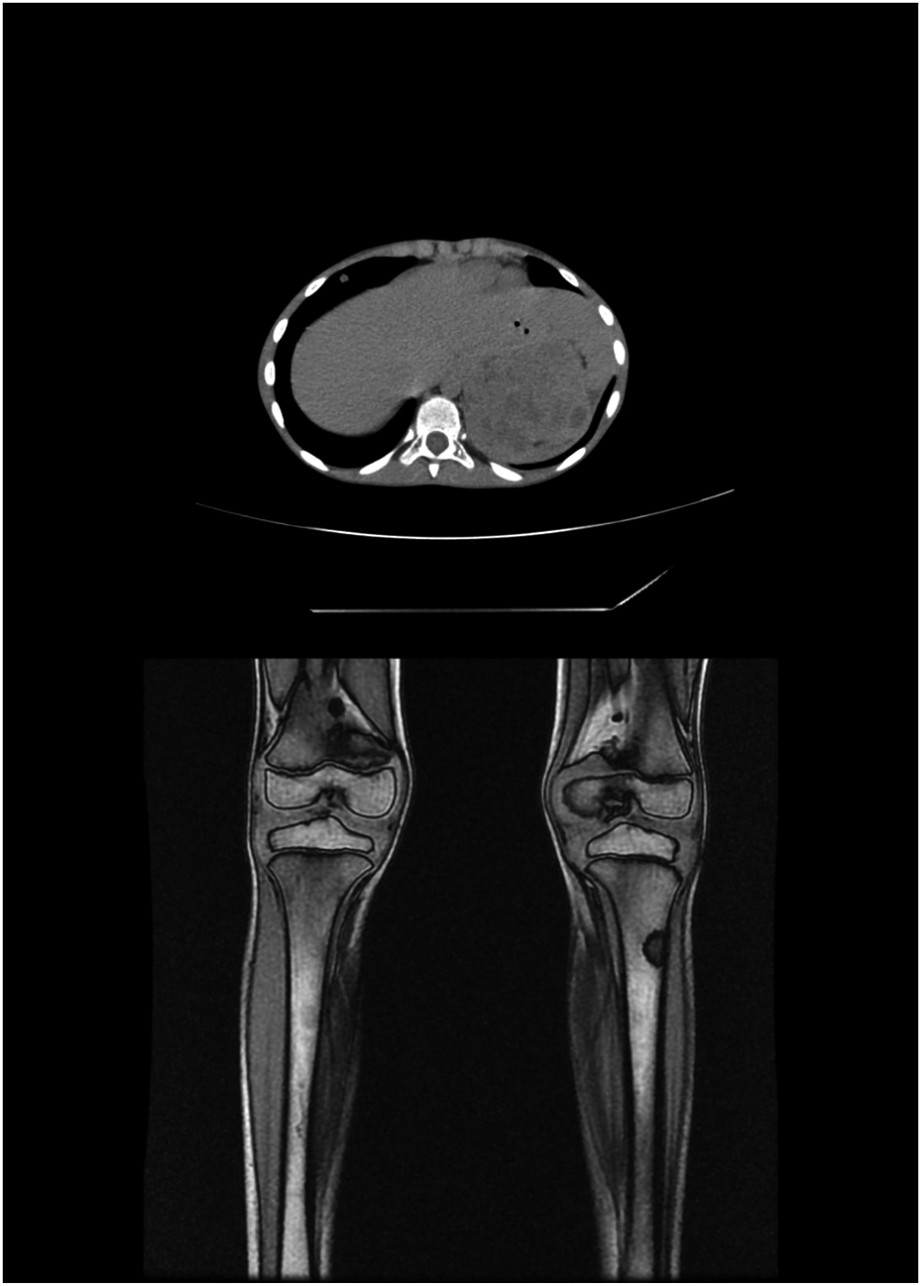
Patient No. 3’s MRI images. MRI, magnetic resonance imaging.

Adrenal glands were the most common primary tumor site in our series and presented as an abdominal mass with symptoms of compression of the abdominal viscera. This finding was consistent with the findings of previous studies, followed by retroperitoneal and pelvic sympathetic ganglia (Figure 2). Thoracic localization was reported in cases studied in the literature, but not in ours. The SEER monograph reported that, regardless of age, neuroblastomas most frequently occurred in the adrenal gland (45).

The benefits of early diagnosis are well documented and include monitoring for malignancy. A possible pathogenic relationship between NF1 and NBT is based on several observations. Neurofibromatosis is a neurocristopathy. The clinical features are pathogenetically united in their origin in neural crest dysgenesis. NBTs arise from cells of the neural crest as well. INSS stage 4s neuroblastoma is characterized by a small primary tumor in infants less than 12 months old in whom remote involvement is confined to the liver, skin, or bone marrow. The tumor can regress to lesions that are pathogenetically indistinguishable from neurofibroma. Early in its development, neurofibroma may cytologically resemble a differentiating neuroblastoma (46). In children, an extremely rapid growth of a neurofibroma is uncharacteristic and warrants investigation for malignancy. In addition to physical examination, imaging studies and urinary catecholamine measurements are indicated. Because management decisions depend on distinguishing malignant from non-malignant tumor, direct tissue diagnosis of both neuroblastoma and neurofibroma is essential.

In the present study, the staging distribution is fairly heterogeneous, with the majority of patients with an INSS stage 4 neuroblastoma. However, it is interesting to note how, compared with the review, the percentage of metastatic tumors has decreased over time. The high percentage of stage 4 in the review study may be attributed to a lack of awareness shown by general practitioners of the probability of cancer, especially in infancy when localized stages are more common, and to the lack of ultrasound use, such that tumors were not initially identified and subsequently regressed or later diagnosed at a more advanced stage in a tertiary hospital. This explanation may also account for similar findings in the postulation by Spix et al., in a study of neuroblastoma in Europe between 1978 and 1992, that the variation in stage distribution between countries may be explained by differences in the frequency of diagnosis of localized cases (5).

In our study, as described by the INSS, the rate of patients with elevated VMA and LDH at diagnosis varies according to the stage, with high-stage tumors being more likely to have pathological values. *MYCN* amplification, performed in five tested tumors, was absent, while the literature refers to only one case in 2003 with amplified *MYCN* (42).

In general, the treatment of neuroblastoma in NF1 patients is similar to that in patients without NF1. When dealing with biopsy specimens, the main concern is related to arriving at a diagnosis. The material should, therefore, be subdivided into at least two parts: one for diagnosis and the definition of cellular composition and the other for touch preparations and molecular biology investigations. In our series, only one patient (No. 4) did not undergo surgery, and diagnosis was made by using bone marrow aspiration; on the remaining patients, biopsy was performed. In our series, multiple-agent chemotherapy was adopted systematically and judged, and it was preferable to invasive surgery as an initial approach. This had numerous advantages for the patients, because no major surgical complications were reported and tumor shrinkage after chemotherapy allowed delayed surgery to be less aggressive. Unfortunately, complete tumor resection could be still difficult to achieve, but our chemotherapy and radiotherapy may be enough to control postoperative residuals, and some patients were cured without having to undergo any major surgery.

An interesting presentation is that of one of our patients (patient No. 5), who showed the most significant number of complications related to NF1, with bilateral hydronephrosis (with nephrostomies), clitoral hypertrophy, pelvic plexiform neurofibromas, neurofibromas with invasion of the spinal canal and compression of the spinal cord, and MPNST. This patient developed a ganglioneuroma, which, as already mentioned, is considered benign (47) and the prognosis is excellent, even when complete tumor removal is not possible. Nevertheless, the patient had a pelvic mass infiltrating the uterus, urethra, and sciatic nerve, with the nodules reaching the right gluteus. The histological examination carried out following biopsy revealed a diagnosis of plexiform neurofibromas with ganglioneuroma areas. Given the extent of the disease and the infiltration of the pelvic organs, surgical treatment was excluded, and it was decided to perform a strict radiological clinical follow-up. Because of the dimensional increase in the mass, the appearance of pain, and the accentuation of itching, the patient was subjected to metabolic radiotherapy with MIBG, given the intense capture of the documented mass with MIBG scintigraphy. After two cycles with no response, attempts were made with different chemotherapy cycles, first with cisplatin and etoposide, then with cyclophosphamide, and finally with methotrexate and vinorelbine. Finally, the patient showed a steady state of the mass with the administration of lenalidomide. Unfortunately, however, the patient died subsequently following the development of a mediastinal MPNST. This presentation should be regarded as an exception.

The analysis of our series of cases shows a death outcome in half of the patients. In the literature review, only two of ten patients reported survived after treatment.

The results indicate that the survival rates reported in our series are higher than those described in previously published reports. For this, many explanations should be considered: the small number of patients in both series, that is, in ours and in the literature; most of our patients have been diagnosed later than those in the literature. Moreover, this may be due to the systematic use of a multidisciplinary approach in all the patients concerned and to the ongoing trials that are exploring the efficacy of new drugs and novel immunological approaches in order to save a greater number of these patients. Unfortunately, the need for an aggressive treatment approach can also mean severe side effects, and this issue should be addressed when planning future treatments.

The current study has several limitations. It is a retrospective study. Compared with the reported national studies in other countries, the relatively small sample size in ours was an important limitation affecting statistical power. However, this is already the largest study on neuroblastoma in patients with NF1, and considering the fact that we do not have a registry on malignancies in NF1 patients, these are the best currently available data.

Undoubtedly, case reports will not have as much potential impact on the science or practice of healthcare as a randomized controlled trial or other research projects on cancerous diseases. However, case reports have a role to play only in evidence-based medicine and constitute the first line of evidence, also considering that in cancer therapies, the number of patients required to conduct such studies cannot always be recruited. It may be the only way to make others in the field aware of unusual presentations or complications, and it is a time-honored vehicle for teaching others.

## Data Availability

The raw data supporting the conclusions of this article will be made available by the authors, without undue reservation.
